# Analysis of Azathioprine Metabolites in Autoimmune Hepatitis Patient Blood—Method Development and Validation

**DOI:** 10.3390/ijms252011233

**Published:** 2024-10-18

**Authors:** Andrea Guba, Patrícia Kováts, Zoltán A. Mezei, Mária Papp, Éva Csősz, Gergő Kalló

**Affiliations:** 1 Proteomics Core Facility, Department of Biochemistry and Molecular Biology, Faculty of Medicine, University of Debrecen, Egyetem tér 1, 4032 Debrecen, Hungary; guba.andrea@med.unideb.hu (A.G.); cseva@med.unideb.hu (É.C.); 2 Metabolomics Research Group, Department of Biochemistry and Molecular Biology, Faculty of Medicine, University of Debrecen, Egyetem tér 1, 4032 Debrecen, Hungary; 3 Doctoral School of Molecular Cell and Immune Biology, University of Debrecen, Egyetem tér 1, 4032 Debrecen, Hungary; 4 Division of Gastroenterology, Department of Internal Medicine, Faculty of Medicine, University of Debrecen, Nagyerdei krt. 98, 4032 Debrecen, Hungary; kovats.patricia@med.unideb.hu (P.K.); papp.maria@med.unideb.hu (M.P.); 5 Kálmán Laki Doctoral School, University of Debrecen, Egyetem tér 1, 4032 Debrecen, Hungary; 6 Institute of Gastroenterology, European Reference Network on Hepatological Diseases, ERN RARE-LIVER, Clinical Center, 4032 Debrecen, Hungary; 7 Department of Laboratory Medicine, Faculty of Medicine, University of Debrecen, Nagyerdei krt. 98, 4032 Debrecen, Hungary; mezeiza@med.unideb.hu

**Keywords:** azathioprine, 6-thioguanine, 6-methyl-mercaptopurine, LC–MS, validation

## Abstract

Autoimmune hepatitis (AIH) is a chronic inflammatory liver disease treated by steroids and immunomodulator thiopurine drugs such as azathioprine (AZA). AZA is metabolized in the human body into bioactive forms such as 6-thioguanine (6-TG) and 6-methyl-mercaptopurine (6-MMP). Monitoring the levels of bioactive AZA metabolites is very important for proper treatment of patients. In this study, our aim was to develop and validate a fast and sensitive ultra-high performance liquid chromatography–mass spectrometry (UHPLC–MS) method for the analysis of 6-TG and 6-MMP from blood samples of patients with AIH to monitor the level of these bioactive metabolites. The detection and quantification of the analytes was carried out by Selected Reaction Monitoring (SRM)-based targeted mass spectrometry. The method was validated according to the EMA guidelines. Blood samples from patients with AIH treated with AZA were analysed with the developed method. The method was successfully validated with appropriate accuracy and precision for the target biomolecules and their concentration in the samples from patients with AIH was determined. The developed and validated UHPLC–MS method enables the fast and precise analysis of AZA metabolites.

## 1. Introduction

Autoimmune hepatitis (AIH) is a rare and chronic inflammatory liver disease that affects primarily women but may occur in all genders at all ages [1]. AIH is characterized by elevated levels of serum immunoglobulin G and circulating autoantibodies, increased activity of transaminases and liver inflammation [1]. In order to prevent the development of recurrent liver diseases such as cirrhosis, acute hepatitis and end-stage liver disease, AIH therapy requires lifelong treatment in the case of most patients [1]. In general, the initial treatment starts with steroid monotherapy, followed by an effective maintenance treatment with azathioprine (AZA) [2,3]. While long-term steroid therapy is associated with several side effects, such as diabetes mellitus, hypertension and weight gain, the incidence of these symptoms can be reduced by a combined therapy of AZA and steroids [1,2,3].

The metabolism of AZA is complex and involves several enzymatic pathways that produce active, inactive and potentially toxic metabolites. Approximately 88% of AZA is converted to 6-mercaptopurine and methylnitro-thioimidazole in red blood cells (RBCs) by gluthatione-S-transferase after the prodrug absorption from the gastrointestinal tract. Furthermore, 6-mercaptopurine can be metabolized via several competing pathways: methylation by thiopurine methyltransferase (TPMT) to 6-methyl-mercaptopurine riboside (6-MMPr) (with potential hepatotoxicity); conversion by xanthine oxidase (XO) to inactive 6-thiouric acid; or conversion by hypoxanthine-guanine phosphoribosyltransferase (HGPRT) to thioinosine monophosphate (TIMP) [4]. Thereafter, TIMP is subsequently converted to 6-thioguanine nucleotides (6-TGNs), which are considered main therapeutic metabolites of AZA/6-mercaptopurine [5]. However, high levels of 6-TGNs can cause life-threatening myelosuppression [4,5].

Apart from AIH treatment, AZA and its metabolites are used for the treatment of chronic inflammatory diseases, childhood acute lymphoblastic leukaemia (ALL) and inflammatory bowel disease (IBD) [6,7,8]. They were first developed for the treatment of leukaemia in paediatric patients, but have been successfully used to treat autoimmune diseases and to prevent graft rejection following solid organ transplantation due to their observed antiproliferative effects on T cells. However, the exact molecular mechanism of the immunosuppressive effect of thiopurines is not well understood [5]. On the other hand, thiopurine drugs can interfere with DNA synthesis by their antagonistic effect with endogenous purines [5]. Thiopurine drugs are effective in the prevention of disease recurrence but not in remission induction [5]. In combination with biological drugs such as anti-tumour necrosis factor (TNF) agents, they have a beneficial effect by improving clinical remission and mucosal healing rates [9]. Patients who develop antibodies against a single anti-TNF drug are much more likely to develop antibodies against a subsequent anti-TNF treatment, and in these cases, therapeutic drug monitoring (TDM) during the combination therapy can be useful for the prevention of immunogenicity to a second anti-TNF therapy. When TDM is applied in anti-TNF and thiopurine combination therapy, the optimization of the therapy should not rely only on monitoring the level of the biological agent, but also on monitoring the level of 6-TG and optimizing the thiopurine concentration in order to maximize the biological effects [10]. Optimizing thiopurine levels through dose modification or pharmacological manipulation is more cost-effective than increasing the dose of the biological drug [10].

AZA requires 2–4 weeks to reach a steady state level, which is a longer time compared to other immunosuppressants [4]. Laboratory monitoring of AZA treatment includes complete blood count (CBC) and liver function tests for hepatitis and leukopenia. Monitoring is recommended every two weeks for the first two months of AZA treatment and every three months during further therapy. The British Gastroenterological Society has recently recommended that thiopurine metabolites should also be monitored during AZA treatment of IBD to prevent toxicity and inappropriate dosing [4]. However, most of the data were obtained from IBD-related studies and no recommendations were made for patients with AIH. Thiopurine metabolites can be monitored by measuring the concentration of 6-TGNs and 6-MMP in erythrocytes. Thereafter, the dose of AZA can be adjusted, to maintain a normal therapeutic range of both 6-TGNs and 6-MMP. This is particularly recommended for patients who have an inadequate response to therapy or have toxic metabolites. However, the monitoring of thiopurine metabolites is only a complementary analysis to standard laboratory measurements [4].

TDM of the active metabolites of AZA is a priority for individualised treatment [11]. In this paper, we report a targeted liquid chromatography–mass spectrometry (LC–MS) method for the detection and quantification of 6-TG and 6-MMP in red blood cells. From these results, the concentration of 6-TGNs and 6-MMPr can be calculated and can be used for TDM in patients with AIH treated with AZA.

## 2. Results and Discussion

In order to monitor the concentration of the selected AZA metabolites in patients with AIH, we have developed a Selected Reaction Monitoring (SRM)-based LC–MS method for the identification and quantification of 6-TG and 6-MMP. As compared to UV detection, mass spectrometry (MS) analysis offers higher selectivity [12]. Complex biological samples contain many organic and inorganic compounds co-eluting with the target analytes. Co-elution can be handled by the optimization of chromatography settings [13,14]; however, the total separation of all compounds in a complex sample is often impossible. SRM experiments rely on the monitoring of specific m/z transitions, thus providing highly selective and specific identification and quantification of the target molecules [15] without the interference of co-eluting compounds.

**Figure 1:**
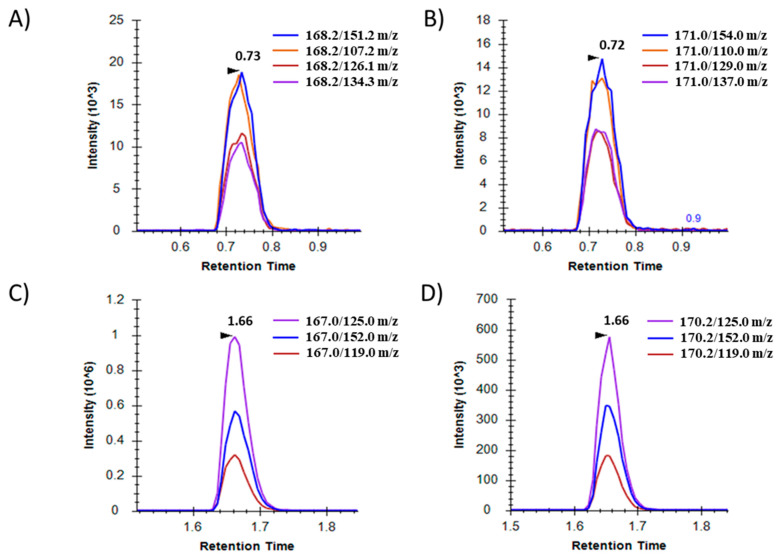
Representative SRM spectra of examined azathioprine metabolites: (**A**) 6-TG, (**B**) 6-TG_IS, (**C**) 6-MMP, (**D**) 6-MMP_IS. The x-axis shows the retention time in minutes while the y-axis shows the peak intensity. The individual SRM transitions are shown as well.

The method was developed on an Acquity H-class ultra-high performance liquid chromatography (UHPLC) system (Waters, Milford, MA, USA) coupled with a 5500QTRAP tandem mass spectrometer (Sciex, Framingham, MA, USA). The elution profile was optimized and a rapid 4 min gradient was developed. The MS analysis was carried out by SRM acquisition according to the gold standard [15]. Stable isotope-labelled internal standards (IS) were used as quality controls and for accurate quantification (6-TG_IS and 6-MMP_IS). The applied SRM transitions were specific for the target analytes and the identification of the target molecules and their synthetic counterparts was done successfully. The observed retention times were 0.73 min (6-TG and 6-TG_IS) and 1.66 min (6-MMP and 6-MMP_IS) (Figure 1).

The developed LC–MS method was further validated according to the guidelines of the European Medicines Agency (EMA) [16]. The selectivity, specificity, carry-over, linear range, lower limit of detection (LLOD), lower limit of quantification (LLOQ), accuracy, precision, recovery, matrix effect and stability were tested.

### 2.1. Sensitivity, Calibration Curve and Range

Blood samples were collected from healthy individuals and their serum was applied as matrix. In the matrix, dilution series of 2.5–2500 ng/mL for each analyte were tested in two parallel runs consisting of the following points: 2.5, 5, 10, 20, 39, 78, 156, 312.5, 625, 1250, and 2500 ng/mL; they were found to have a linear range of 5–1250 ng/mL. To determine the calibration curve, LLOQ and ULOQ, the linear range was investigated in three independent runs and two replicates over three days. Linear regression analysis was performed using a logarithmic scale and the linear equations were y = 0.0073*x + 0.0042 (R^2^ = 1) and y = 0.0087*x − 0.0035 (R^2^ = 0.9993) for 6-TG and 6-MMP, respectively. LLOQ was 5 ng/mL and ULOQ was 1250 ng/mL for each analyte (Table S1). Our measurement data conform to the EAM guidelines: the accuracy of each calibration standard is less than 20% of the nominal concentration for the LLOQ value and less than 15% for all other levels [16].

### 2.2. Selectivity, Specificity and Carry-Over

In order to determine the selectivity and carry-over of the applied system, six individual blank samples (matrix samples without any analyte and IS) were used. In the case of selectivity of 6-TG, the analyte response at the LLOQ was 7.6%, while in the case of 6-MMP it was 2.31%; the internal standard responses at the LLOQ were 1.12% and 0.02%, respectively (Table S2).

During the analysis of the carry-over, the analyte responses for 6-TG and 6-MMP in the blank sample after ULOQ injection were 10.37% and 3.28%, respectively, while the IS responses were 1.09% and 0.01%, respectively, (Table S2) in compliance with the EMA guidelines [16].

Specificity was tested by using 5-bromouracil (100 ng/mL) as a potential interfering compound. Our results showed that the analyte responses were 5.17% and 1.48% in 6-TG and 6-MMP, respectively, while the IS responses were 0.15% and 0.03% in 6-TG_IS and 6-MMP_IS, respectively, in the studied retention time ranges (Table S2). All of our registered data were below 20% and 5% for the analyte and IS responses, respectively, which is in accordance with the EMA guidelines [16].

### 2.3. Accuracy, Precision and Recovery

During method validation, the accuracy, precision and recovery of intraday and interday runs were examined (Table 1).

Our data show that the accuracy, recovery and precision (CV%) at each concentration were less than 15% of the nominal concentration. The results indicate that our method fulfilled the EMA validation criteria [16].

### 2.4. Matrix Effect

The matrix effect was examined by the analysis of three replicates of low concentration (20 ng/mL) and high concentration (1250 ng/mL) QCs (internal standards spiked into serum samples) from 6 different batches. The results of the matrix effect analyses are shown in Table 2.

The precision (CV%), the recovery and the matrix effect at each concentration were found to be less than 15% of the nominal concentration. The obtained results are in accordance with the EMA criteria [16].

### 2.5. Stability

During validation, the freeze–thaw stability, long-term storage (10 weeks) stability and the stability of the analytes in the working solutions at −20 °C were examined. The stability of the analyte in the matrix was evaluated by the analysis of two replicates of low (20 ng/mL) and high (1250 ng/mL) concentration of QCs, and the results are shown in Table 3.

We have also tested the autosampler stability by the analysis of six samples from patients with AIH treated with AZA right after the sample preparation and after storage for 6 h in the autosampler (4 °C). The results are shown in Table 4.

Reinjection reproducibility was assessed by reinjecting five replicates of the low, middle and high QCs after storage in the autosampler for 12 h and then at −20 °C for three hours (Table 5).

Considering recovery, the differences in the 6-TG case were lower than 15% and in the 6-MMP case were between 25% and 60%. The highest recovery was measured in the 20 ng/mL concentration case.

Our results indicate that for short and long term stability, the results are within the limits given by the EMA guidelines [16]. Although, three freeze–thaw cycles did not change the concentration of the target molecules, the concentration in the samples stored at −20 °C showed a significant decrease over the studied time period (10 weeks). These data suggest that long term storage of the samples should be avoided. The 6 h exposure in the autosampler did not change the concentration of the analytes. However, when the re-injection reproducibility of the samples was investigated, a much larger difference was obtained, especially for low concentrations and 6-MMP.

### 2.6. Analysis of the Concentration of AZA Metabolites in Samples from Patients with AIH

In order to demonstrate that our method is suitable for the analysis of the concentration of 6-TG and 6-MMP in complex biological samples with potential interfering agents, blood samples from patients with AIH treated with AZA (Imuran, Aspen Pharma Trading Ltd., uMhlanga, Republic of South Africa) were processed and analysed. The concentrations determined by our method were further used to calculate the concentration of 6-TGNs and 6-MMPr in the blood samples of the subjects by normalization of the RBC count [17,18], using the formula presented in Section 3.6. All target analytes were identified and quantified in the examined blood samples, and the RBC count normalized amounts were calculated (Table 6).

Our data show that the quantification of 6-TG and 6-MMP in the blood samples of patients with AIH can easily be done using the developed and validated LC–MS method. The analysis of the concentration of AZA metabolites during the treatment is important to prevent drug-related toxicity. A high level of 6-MMPr was found to be correlated with hepatotoxicity; patients with >5700 pmol/8 × 10^8^ RBC level of 6-MMPr have a three-fold increased risk of hepatotoxicity [5,19,20]. The analysis of the level of 6-TGN can be useful for the identification of patients who do not respond to the therapy despite the optimal AZA/6-mercaptopurine dose [21].

Recently Yu et. al. presented a UPLC–UV method for the determination of 6-TG and 6-MMP from red blood cells [7]. Although, their method proved to be useful for the analysis of AZA metabolites, our method utilizes targeted MS with stable isotope-labelled standards, offering higher precision and selectivity. The linear dynamic range of our method is slightly narrower than the method developed by Yu et. al., the LLOQ for both analytes is lower because of the higher sensitivity of the MS analysis. It also should be highlighted that the MS-based target identification provides very high selectivity as the potential co-eluting interfering agents can be excluded. The UHPLC–MS method developed by our group is suitable for the reliable examination of blood samples from patients having a low concentration of circulating AZA metabolites.

A possible limitation of our study is that we have focused on the analysis of only two AZA metabolites (6-TG and 6-MMP), but the analysis of other potential metabolites (such as 6-thiouric acid) or potential toxic derivates (such as 6-thioinosine-monophosphate) was not included. Another limitation might be the applied instrumentation; however, the selectivity provided by the mass spectrometers in discriminating the target analyte from the potential co-eluting interfering agents makes their utilization imperative, especially when important—such as therapy-related—decisions have to be made.

In this article, we have presented a fast and sensitive, validated UHPLC–MS method for the analysis of two AZA metabolites, 6-TG and 6-MMP. The method was found to be accurate, sensitive and precise and meets the EMA criteria. We also tested the method on blood samples from patients with AIH treated with AZA. The results indicated that the developed assay is suitable for the quantification of the target metabolites in clinical samples and can help to adjust the dose of the drug during the treatment.

## 3. Materials and Methods

### 3.1. Chemicals and Reagents

HPLC-grade methanol, acetonitrile and water were obtained from VWR Ltd. (Radnor, PA, USA). HPLC grade trifluoroacetic acid, 6-thioguanine (6-TG), 5-bromouracil and dl-dithiothreitol (DTT) were purchased from Sigma-Aldrich (St. Louis, MO, USA). 6-methyl-mercaptopurine (6-MMP) and internal standards (IS) 6-thioguanine-^13^C^15^N (isotopic purity 98 atom% ^13^C, 98 atom% ^15^N) and 6-methyl-mercaptopurine-D_3_ (isotopic purity 98 atom% D_3_) were purchased from Toronto Research Chemicals Inc. (Toronto, ON, Canada).

### 3.2. Instruments and Software

To conduct the analyses, a Waters ACQUITY H Class ultra-performance liquid chromatography system controlled by the Empower 3 software (Waters, Milford, MA, USA) coupled with a 5500 QTRAP (Sciex, Framingham, MA, USA) mass spectrometer controlled by the Analyst software (version 1.6.3, Sciex, Framingham, MA, USA) was used. MS data were processed using the Skyline software (version 23.1.0.455) [22]. The red blood cell counts were done by the Sysmex XN-2000 automatic haematology analyser (Sysmex Corporation, Kobe, Japan) [23].

### 3.3. Chromatographic and Mass Spectrometric Conditions

The liquid chromatographic separation of the targeted molecules was carried out on an AccQ-tag Ultra C18 column (1.7 µm; 2.1 × 100 mm, Waters, Milford, MA, USA) guarded by an Acquity in-line filter (0.2 µm; 2.1 mm Waters, Milford, MA, USA). The whole analysis was conducted in 4 min including re-equilibration with a 0.40 mL/min eluent flow rate and a 40 °C column temperature. Mobile phase A was 0.02 mol/L ammonium formate with 0.3% (*v*/*v*) formic acid in water (pH = 3.00), and mobile phase B was 100% acetonitrile. Table 7 contains the elution gradient of the UPLC separation.

SRM-based targeted MS analysis was performed using electrospray ionization in positive ion mode with a 5500 V spray voltage. The source temperature was set to 500 °C, ion source gas 1 was set to 30 psi and ion source gas 2 to 50 psi; the curtain gas was 30 psi. The applied declustering potential was 100 eV, and a 40 eV collision energy was used for fragmentation. The stable isotope-labelled compounds 6-thioguanine-^13^C^15^N (6-TG_IS) and 6-methyl-mercaptopurine-D_3_ (6-MMP_IS) were used as internal standards (IS). The detailed parameters of the SRM experiment are shown in Table 8.

The SRM spectra were analysed with the Skyline software (version 23.1.0.455) [22]. The acquired SRM data were uploaded to the Panorama [24] website (https://panoramaweb.org/University%20of%20Debrecen/AZA/project-begin.view (accessed on 20 September 2024)) and are publicly available.

### 3.4. Preparation of QC Samples and Standard Solution

The standard solution of the AZA metabolites (6-TG and 6-MMP) was prepared in methanol-2M NaOH (3:7) for 6-TG and 6-TG_IS and in methanol for 6-MMP and 6-MMP _IS in a 1 mg/mL final concentration. The individual solutions were stored at −20 °C. The calibration standards were prepared from the standard solutions through serial dilutions with methanol–water (3:7). The QC samples were prepared in serum obtained from healthy volunteers and spiked with the standard solution of 6-TG and 6-MMP. The final concentration of each analyte was 5 ng/mL, 20 ng/mL, 625 ng/mL and 1250 ng/mL, respectively. QC samples were used for the analysis of the recovery, matrix effect, intra- and inter-day precision and stability.

### 3.5. Method Validation

The developed LC–MS method was validated according to the European Medicines Agency (EMA) ICH guideline M10 on bioanalytical method validation and study sample analysis [16], as described by Yu et al. and Miao et al. [7,11]. The linearity, selectivity, specificity, accuracy and precision, intra- and inter-day variability, carry-over, recovery, matrix effect, lower limit of quantification (LLOQ) and upper limit of quantification (ULOQ) were determined.

The calibration standards were prepared in the same biological matrix as the study samples. The calibration range was defined by the LLOQ, which is the lowest calibration point, and the ULOQ which is the highest calibration point. Calibration curves of 2.5–5000 ng/mL range were tested in the matrix according to the EMA guidelines. The criteria were the following: at the LLOQ, the accuracy of each analyte should be within ±20% of the nominal concentration; at all the other levels the accuracy should be within ±15%; and at least 75% of the calibration series should fulfil the criteria [16].

In order to determine the selectivity, blank samples were analysed without the addition of the analytes or ISs. The criteria were that there should be no observed interfering peaks at the studied retention times.

Specificity was determined using 5-bromouracil, a purine analogue of the studied analytes that should not interfere with the target analytes and ISs.

The accuracy and precision were determined by analysing QC samples in 5, 20, 625 and 1250 ng/mL concentrations. Intra- and inter-day variability was determined by the analysis of five replicates of the QC samples in the same day (intraday) and for 2 days.

The carry-over of the system was tested by the analysis of blank samples after the ULOQ of the calibration curves.

The matrix effect was examined by the analysis of three replicates of low concentration (20 ng/mL) and high concentration (1250 ng/mL) QCs from 6 different matrix batches compared with same concentration in water-based solution (methanol–water (3:7)). The matrix effect is defined as an alteration of the analyte response due to interfering and unidentified components in the sample matrix. We used the following formula to calculate the matrix effect (ME):

ME = [(Cw − Cm)/Cw] × 100,(1)

where Cw is the concentration of the analytes in MilliQ water and Cm is the concentration of the same concentration of the analytes spiked into matrix. The accuracy and coefficient of variation should not be more than 15%.

The freeze–thaw stability of the samples was tested by the analysis of the QC samples after three freeze–thaw cycles at −70 °C according to the EMA guidelines with the following criteria: low and high QC samples (20 ng/mL and 1250 ng/mL, respectively) should be thawed and analysed according to the same procedures as the test samples. QCs should be kept frozen for at least 12 h between thawing cycles. Freeze–thaw stability of QCs should be performed using freshly prepared calibration standards and QCs. The number of validated freeze–thaw cycles shall be at least three. We have also tested the long-term stability of the analytes in matrix stored in at −20 °C for 10 weeks.

The stability of the stock solutions and working solutions of the analyte and IS was determined under the storage conditions used for the analysis of the test samples by using the lowest and highest concentrations of the solutions. The stability of the processed samples, including the time to completion of the analysis, and the stability of the processed sample in the autosampler was determined as well. Reinjection reproducibility was also tested.

### 3.6. Preparation and Analysis of Blood Samples

Blood samples from AIH patients treated with AZA-containing Imuran (Aspen Pharma Trading Ltd., uMhlanga, Republic of South Africa) were collected by the members of the Department of Gastroenterology, Faculty of Medicine, University of Debrecen. Sample collection complied with the guidelines of the Helsinki Declaration and ethical approval was obtained from the University of Debrecen Ethics Committee (12759-6/2019/EÜIG).

EDTA-treated whole blood samples were lysed by mixing them with two volumes of UPLC-grade water for 1 min. Then, 200 µL lysate was mixed with 100 µL of IS (0.2 µg/mL for each compound) and with 150 µL (0.1 mol/L) DTT in water. Proteins were precipitated by mixing the samples with 40 µL 100% trifluoroacetic acid for 1 min and the samples were further centrifuged for 15 min with 16,100× *g*. Then 2 × 200 µL supernatant was transferred into a new tube and incubated at 100 °C for 45 min followed by a centrifugation for 15 min with 16,100× *g*. Thereafter, 200 µL supernatant was transferred into a new tube and centrifuged again with the same conditions. A volume of 100 µL of clear supernatant was transferred into sample vials and 5 µL was injected in duplicates to the UPLC–MS system. 6-TGNs were hydrolysed to 6-TG by heating under acidic conditions, and the concentration of 6-TGNs was determined by the analysis of the level of 6-TG.

The concentrations of 6-TG and 6-MMP were presented in ng/mL units, and the concentration of 6-TGNs and 6-MMPr were calculated by normalization on the RBC count [18] using the following formula:

C (pmol/8 × 10^8^ RBC) = 8 × 100 × C (ng/mL)/M (g/mol)/RBC (T/L), (2)

where C (pmol/8 × 10^8^ RBC) stands for concentration in pmol/8 × 10^8^ RBC, C (ng/mL) stands for concentration of analyte in ng/mL, M (g/mol) for molecular weight of the analyte, and RBC (T/L) stands for red blood cell count in 10^12^/L in EDTA-treated whole blood samples.

## 4. Conclusions

We have presented a validated fast and sensitive UHPLC–MS method for the simultaneous determination of 6-TGNs and 6-MMPr azathioprine metabolites in human blood. This new method is characterized by a fast turnaround time of 4 min with equilibration, which is shorter than most LC–MS/MS methods. In addition, this assay has shown good sensitivity and stability and can be used for routine TDM for azathioprine treatment in AIH patients. The presented method can be also used as a starting point for further method development to monitor the concentration of other AZA metabolites to cover the whole bioprocessing pathway in the treated patients.

## Figures and Tables

**Table 1:** Accuracy, precision and recovery of 6-TG and 6-MMP.

Analyte	Nominal Concentration (ng/mL)	Intraday (n = 5)				Interday (n = 15)			
		Mean (ng/mL)	SD	CV%	Recovery%	Mean (ng/mL)	SD	CV%	Recovery%
6-TG	5	4.49	0.19	4.22	89.72	4.31	0.63	14.55	86.18
	20	19.34	1.01	5.23	96.71	17.82	2.42	13.60	89.12
	625	605.36	15.05	2.49	96.86	626.49	53.68	8.57	100.24
	1250	1171.08	21.74	1.86	93.69	1252.11	122.61	9.79	100.17
6-MMP	5	5.07	0.38	7.44	101.40	4.69	0.64	13.66	93.86
	20	18.88	0.87	4.63	94.40	18.00	2.69	14.93	90.00
	625	598.71	16.06	2.68	95.80	667.39	73.10	10.95	106.78
	1250	1096.89	19.03	1.73	87.75	1207.29	179.60	14.88	96.58

**Table 2:** The matrix effect and recovery for the determination of 6-TG and 6-MMP.

Analytes	QC (ng/mL)	Mean Conc. (ng/mL)	SD	CV% (RSD%)	Recovery (%)	Matrix Effect (%)
6-TG	20	20.38	2.43	11.93	101.89	5.03
	1250	1436.80	175.36	12.20	114.94	−12.79
6-MMP	20	22.28	3.21	14.42	111.42	3.08
	1250	1436.06	212.73	14.81	114.88	−12.88

**Table 3:** Summary of the results of stability tests of 6-TG and 6-MMP.

Analyte		6-TG		6-MMP	
QC (ng/mL)		20	1250	20	1250
After three freeze–thaw cycles	Mean (ng/mL)	21.42	1439.19	22.65	1240.67
	SD	2.11	22.00	0.01	14.01
	CV%	9.84	1.53	0.02	1.13
	Recovery%	107.11	115.13	113.25	99.25
Long-term storage at −20 °C	Mean (ng/mL)	12.86	822.29	13.87	764.15
	SD	0.14	16.05	0.04	2.35
	CV%	1.09	1.95	0.30	0.30
	Recovery%	64.29	65.78	69.35	61.13
Working solution stability at −20 °C	Mean (ng/mL)	26.17	1583.54	20.41	1369.72
	SD	1.65	75.22	0.20	10.70
	CV%	6.29	4.75	0.97	0.78
	Recovery%	130.87	126.68	102.05	109.58

**Table 4:** Summary of the results of autosampler stability test of 6-TG and 6-MMP in blood samples.

Analyte	Subject Code	Concentration (ng/mL)	Concentration (ng/mL)	SD	CV%
		0 h	6 h		
6-TG	S_06	73.36	74.93	3.71	5.00
	S_09	81.13	80.03	2.64	3.28
	S_26	111.40	116.84	6.38	5.59
	S_31	103.80	99.95	3.55	3.49
	S_38	69.82	71.53	1.82	2.58
	S_58	92.72	92.17	1.47	1.59
6-MMP	S_06	2631.37	2594.92	22.36	0.86
	S_09	1929.34	1859.15	67.35	3.56
	S_26	877.86	931.80	51.64	5.71
	S_31	2489.42	2587.77	58.55	2.31
	S_38	451.82	429.45	18.42	4.18
	S_58	805.33	795.90	39.48	4.93

**Table 5:** Summary of the results of reinjection reproducibility tests for 6-TG and 6-MMP.

Analytes	Nominal Conc. (ng/mL)	Mean Observed Conc. (ng/mL)	SD	CV%	Recovery (%)
6-TG	20	22.88	0.19	0.85	114.22
	625	552.88	20.71	3.75	88.46
	1250	1250.95	43.59	3.49	100.08
6-MMP	20	31.85	2.24	7.04	159.27
	625	782.60	58.94	7.53	125.22
	1250	1620.34	26.72	1.65	129.63

**Table 6:** Concentration and RBC count-normalized concentration of AZA metabolites in the blood samples of patients with AIH. RBC (T/L): red blood cell count in 10^12^/L in samples.

Subject Code	AZA (Imuran) Dose (mg)	RBC (T/L)	Concentration (ng/mL)		Concentration (pmol/8 × 10^8^ RBC)	
			6-TG	6-MMP	6-TGNs	6-MMPr
S_34	25.0	4.48	23.82	11.72	25.44	12.60
S_40	100.0	4.08	128.26	2175.13	150.42	2566.01
S_42	50.0	3.24	45.81	69.86	67.66	103.78
S_56	37.5	3.97	132.18	1445.52	159.31	1752.54

**Table 7:** Elution gradient for the analysis of AZA metabolites. Mobile phase A was 0.02 mol/L ammonium formate with 0.3% (*v*/*v*) formic acid in water (pH = 3.00), and mobile phase B was 100% acetonitrile.

Time (min)	Flow Rate (mL/min)	Mobile Phase A (%)	Mobile Phase B (%)	Curve
0.00	0.40	90	10	-
0.10	0.40	90	10	6
1.00	0.40	10	90	6
1.50	0.40	90	10	6
4.00	0.40	90	10	6

**Table 8:** Parameters of the Selected Reaction Monitoring (SRM) analysis. Q1 m/z: Parent ion m/z, Q3 m/z: Fragment ion m/z, t_R_ window: Retention time window in minutes. 6-TG_IS: Stable isotope-labelled 6-thioguanine-^13^C^15^N. 6-MMP_IS: Stable isotope-labelled 6-methyl-mercaptopurine-D_3_.

ID	Q1 Mass (Da)	Q3 Mass (Da)	t_R_ Window (min)
6-TG	168.2	151.2	0.48–1.14
6-TG	168.2	134.3	0.48–1.14
6-TG	168.2	126.1	0.48–1.14
6-TG	168.2	107.2	0.48–1.14
6-TG_IS	171.0	154.0	0.48–1.14
6-TG_IS	171.0	137.0	0.48–1.14
6-TG_IS	171.0	129.0	0.48–1.14
6-TG_IS	171.0	110.0	0.48–1.14
6-MMP	167.0	152.0	1.36–2.02
6-MMP	167.0	134.0	1.36–2.02
6-MMP	167.0	119.0	1.36–2.02
6-MMP_IS	170.2	152.0	1.36–2.02
6-MMP_IS	170.2	125.0	1.36–2.02
6-MMP_IS	170.2	119.0	1.36–2.02

## Data Availability

The acquired SRM data are publicly available at the Panorama website: https://panoramaweb.org/University%20of%20Debrecen/AZA/project-begin.view (accessed on 20 September 2024).

## Supplementary Materials

The following supporting information can be downloaded at: https://www.mdpi.com/article/10.3390/ijms252011233/s1.
